# Role of platelets and platelet receptors in cancer metastasis

**DOI:** 10.1186/s13045-018-0669-2

**Published:** 2018-10-11

**Authors:** Martin Schlesinger

**Affiliations:** Pharmaceutical Institute, University of Bonn, Bonn, Germany

**Keywords:** Platelets, Metastasis, GPVI, FcγRIIa, CLEC-2, ITAM, hemITAM

## Abstract

The interaction of tumor cells with platelets is a prerequisite for successful hematogenous metastatic dissemination. Upon tumor cell arrival in the blood, tumor cells immediately activate platelets to form a permissive microenvironment. Platelets protect tumor cells from shear forces and assault of NK cells, recruit myeloid cells by secretion of chemokines, and mediate an arrest of the tumor cell platelet embolus at the vascular wall. Subsequently, platelet-derived growth factors confer a mesenchymal-like phenotype to tumor cells and open the capillary endothelium to expedite extravasation in distant organs. Finally, platelet-secreted growth factors stimulate tumor cell proliferation to micrometastatic foci. This review provides a synopsis on the current literature on platelet-mediated effects in cancer metastasis and particularly focuses on platelet adhesion receptors and their role in metastasis. Immunoreceptor tyrosine-based activation motif (ITAM) and hemi ITAM (hemITAM) comprising receptors, especially, glycoprotein VI (GPVI), FcγRIIa, and C-type lectin-like-2 receptor (CLEC-2) are turned in the spotlight since several new mechanisms and contributions to metastasis have been attributed to this family of platelet receptors in the last years.

## Background

Besides their crucial role in coagulation and maintaining hemostasis following mechanical injury of the vasculature, platelets contain a plethora of bioactive molecules in their granules and express different receptors on their surfaces that also contribute to inflammation, cancer progression, and metastasis. In the initial minutes, when tumor cells detach from the primary tumor and access the blood, platelets are the first host cells they encounter. The first descriptions of cancer-associated thrombophlebitis date back 1000 years BC and were later on rendered more precisely by Armand Trousseau in 1865 [1, 2]. Formation of tumor cell platelet aggregates were observed in mouse and rat experimental metastasis models; enhanced metastases formation to the lungs was accompanied by thrombocytopenia [3–6]. Subsequent to this early observations of heterotypic and protumorigenic aggregates of tumor cells and platelets, in the last decades, the knowledge on how tumor cells exploit platelets for survival, arrest, and finally extravasation from blood vessels to distant organs has tremendously increased. Thus, various excellent reviews have been dedicated to the role of platelets in cancer metastasis in the last years, discussing the contribution of adhesion receptors like P-selectin, or integrin α_IIb_β_III_, platelet-activating receptors such as P_2_Y_12_ or protease-activated receptor-1 (PAR-1), or platelet-derived growth factors and chemokines in detail [7–10]. In contrast, the three ITAM (immunoreceptor tyrosine-based activation motif)-associated receptors on human platelets, namely CLEC-2, GPVI, and FcγRIIa, have mostly been investigated in course of hemostasis and thrombosis, but their involvement in cancer metastasis has been neglected widely. Thus, this review provides a summary of platelet protumorigenic effects and focuses in particular on recent findings concerning ITAM-affiliated receptors and their impact on tumor cell platelet interaction.

## Role of platelets in cancer metastasis

### Platelet activation

Tumor cells that enter the blood circulation have to cope with high shear rates and the immune surveillance, e.g., the assault of natural killer cells. Only a very small percentage of tumor cells in the circulation ends up in a metastatic foci, making this process very inefficient [11, 12]. Platelets protect circulating tumor cells (CTCs) by encasing tumor cells in a thrombus, protecting them from cytolysis by natural killer cells [13]. For a stable adhesion between platelets and tumor cells, tumor cells activate platelets by distinct mechanisms, which are also the reason for hypercoagulation and increased risks of thrombosis in cancer patients. Tumor cells release soluble mediators like ADP [13, 14], thromboxane A2 (TXA2) [15, 16], or high-mobility group box 1 (HMGB1), which ligates with toll-like receptor 4 (TLR4) to instigate a local platelet activation [17]. Recently, Ward et al. revealed that cancer cell-expressed adhesion GPCR CD97 induced platelet activation which leads to lysophosphatidic acid (LPA) release from platelets. LPA in turn enhances tumor cell invasiveness and vascular permeability to promote transendothelial migration [18]. Some cancer cells express tissue factor (TF) on their cell membranes, which activates the plasmatic coagulation cascade and finally generates thrombin which in turn induces platelet activation [19]. Besides the activation of the coagulation cascade and platelets, thrombin is of key importance for almost every step of the metastatic cascade. Thrombin favors tumor cell proliferation and tumor growth, e.g., by activation of PAR-1 and fibrinogen [20]. In tumor microenvironment, thrombin-stimulated fibroblasts and macrophages secrete monocyte chemotactic protein which fosters protumorigenic myeloid cell invasion [21]. Thrombin has also several effects on endothelial cells that support angiogenesis for example by potentiation of the mitogenic activity of VEGF on endothelial cells [22]. Additionally, thrombin inhibits apoptosis and induces proliferation and differentiation of vascular progenitor cells [23]. Thrombin-stimulated endothelial cells reveal a rounded morphology and loss of adherens junctions which facilitates tumor cell transendothelial migration [24].

Besides thrombin, several other mechanisms were elucidated which exert a proper tumor cell-induced platelet activation. Recently, Shao could reveal that carcinoma mucins initiate a reciprocal activation mechanism in neutrophils and platelets. Mucins bind to P-selectin on platelets and l-selectin on neutrophils bringing both cells in close proximity. A direct interaction of platelet P-selectin and PSGL-1 on neutrophils induces release of cathepsin G from neutrophils inducing platelet activation [25]. Additionally, some tumor patients suffering from various kinds of cancer are deficient in enzymes cleaving von Willebrand factor (vWf), which could finally lead to a platelet activation by highly polymeric forms of vWf in the circulation and enhanced number of metastases [26]. Already in 1981, tumor cell-derived microparticles with proagulant properties could be detected in vitro and in ascitic fluids of tumor bearing guinea pigs [27]. Later on, TF was identified on tumor-derived vesicles as the reason for procoagulant activity [28]. Furthermore, tumor cell-derived matrix metalloproteinases (MMPs) can elicit platelet activation, and platelet-activating factor in turn can induce an increased MMP expression in melanoma cells [29, 30].

Thus, tumor cells possess various different options to induce coagulation and platelet activation in their close vicinity. Subsequent to platelet activation, tumor cells and platelets form aggregates using miscellaneous adhesion receptors. Chew and Wallace could demonstrate a fibrin deposition to the cloak of platelets and tumor cells 5 min after tail vein injection of tumor cells in Sprague-Dawley rats [31]. Fibrin deposition reached a maximum after 1 h and disappeared 9 h after tumor cell inoculation. Accompanied by platelet and fibrin formation around tumor cells in the early phase of tumor cell dissemination, single neutrophils and lymphocytes could also be detected in the growing aggregates.

### Escape the immune surveillance

The heterogenous cloaks of platelets and tumor cells protect tumor cells from high shear forces in the blood circulation and from attack by leukocytes. First, Nieswandt proposed the idea that platelets impact NK cell-mediated lysis of tumor cells using thrombocytopenic mice [13]. Later on, this hypothesis was corroborated by Palumbo et al. utilizing mice deficient in either fibrinogen or G protein α(q) expression, a protein essential for platelet activation. In both approaches, a strong reduction in tumor cell survival was detectable. Interestingly, in mice with a life-long genetic deficit in NK cells, reduction of tumor cell survival in the lungs was not detectable in a concomitant absence of fibrinogen [32–34]. These data indicated that platelets and fibrinogen shield tumor cells in the vasculature from clearance by NK cells. However, the exact mechanism induced by platelets to protect tumor cells from immunosurveillance was ambiguous. Salih and colleagues revealed that the cloak of platelets and tumor cells exhibit an active phenotype, secreting a plethora of platelet-derived factors like Interferon-γ (IFN-γ) or transforming growth factor-β1 (TGF-β1) [35]. TGF-β1 induced a downregulation of C-type lectin-like NKG2D receptors, which caused a reduced antitumor activity in NK cells. Furthermore, Salih demonstrated that platelets confer MHC class I molecules to tumor cell membranes by direct contact formation that leads to alleviated NK cell reactivity and cytotoxicity towards tumor cells [36]. This mechanism of molecular mimicry adapting a pseudonormal phenotype allows tumor cells to downregulate MHC class I molecules to escape T cell immune surveillance. In the blood stream, the transfer of immunoregulatory proteins finally leads to a MHC class I-positive phenotype without induction of NK cell activity. Additionally, due to tumor cell-mediated platelet activation, TNF family member glucocorticoid-induced TNF-related ligand (GITRL) located in α-granules is coexpressed with P-selectin on platelets surface. GITRL showed no effect on platelet activation or function, whereas NK cell cytotoxicity and IFN-γ release is surprisingly reduced. Platelet-derived GITRL seems to activate GITR on NK cells and instigate a tumor cell phenotype with mitigated immunogenicity [37]. Blocking GITR on NK cells partially compensates reduced NK cell cytotoxicity induced by platelet covered tumor cells. Next to NK cells, platelets can also interfere with dendritic cells, neutrophils, macrophages, and lymphocytes and modulate their immunological function [38–41]. Nonetheless, the impact of platelets on adaptive immunity is ambiguous. Recently, Rachidi found that platelets are the major source of TGF-β in the tumor microenvironment by expression of the TGF-β-docking receptor glycoprotein A repetitions predominant (GARP). The function of GARP is to activate latent TGF-β in the close proximity of platelets. The GARP-TGFβ complex together with platelet-secreted lactate inhibited T cell immunity against both melanoma and colon cancer [42, 43]. These data substantiate the notion that platelets interfere with the innate as well as with the adaptive immune system to tackle cancer cells.

**Fig. 1:**
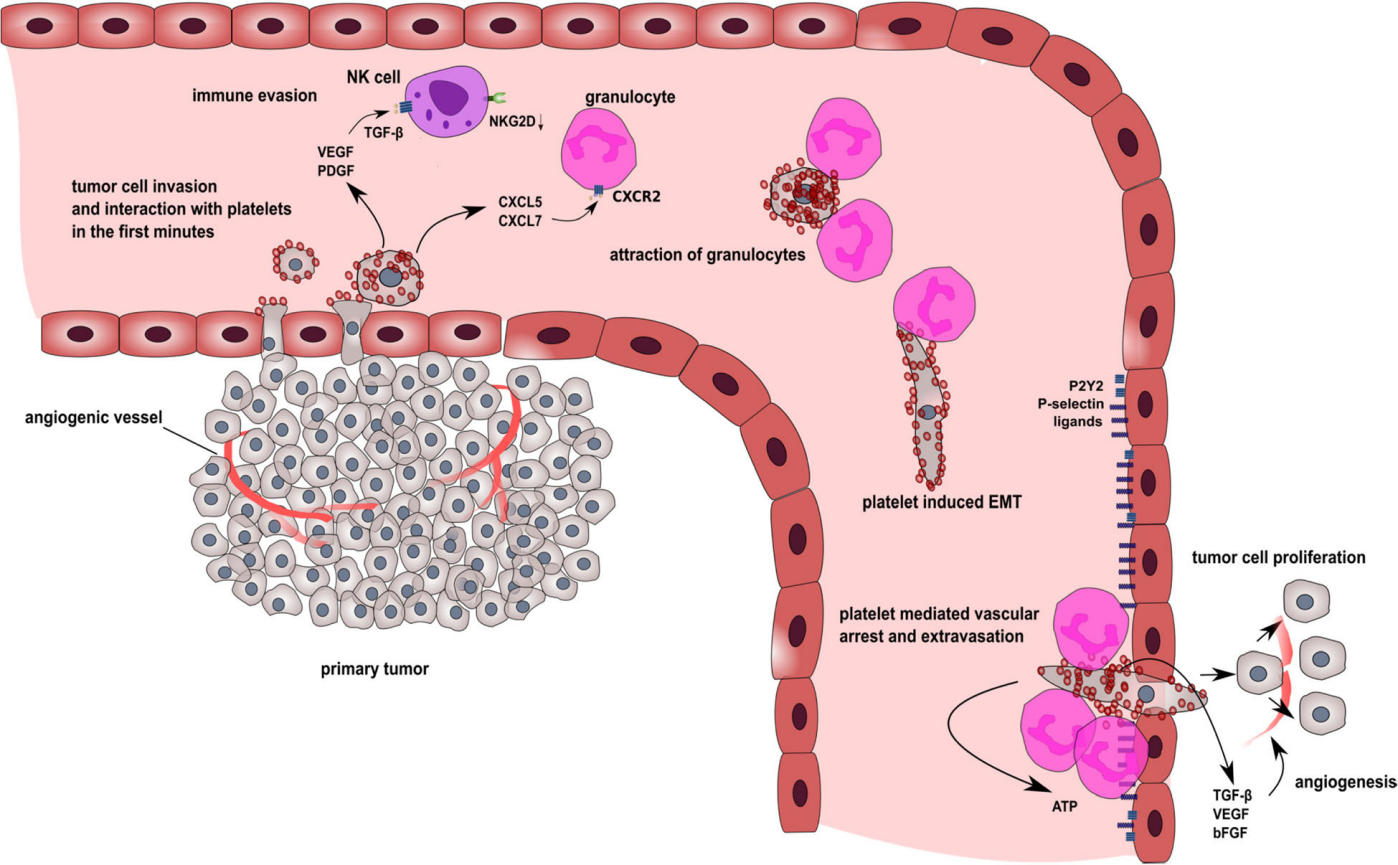
Schematic overview of the metastatic cascade with focus on platelets. Tumor cells detach from the primary tumor and invade the blood circulation. Tumor cells immediately activate and are encased by platelets. Platelets secrete a plethora of growth factors and chemokines (like VEGF, PDGF, or TGF-β) upon activation which induce NK cell anergy due to NKG2D downregulation. In addition, granulocytes are attracted to the agglomerate of platelets and tumor cells by chemokines CXCL5 and CXCL7. Furthermore, platelets are capable to shift tumor cell phenotype from an epithelial to a mesenchymal-like. Finally, platelets mediate tumor cell arrest at the vascular wall via P-selectin and its ligands and facilitate tumor cell extravasation to the subendothelial matrix of a distant organ by activation of endothelial P_2_Y_2_ receptor. In order to establish a metastatic foci, platelet-derived growth factors, e.g., TGF-β, VEGF, or bFGF instigate tumor cell proliferation and neovessel formation

Platelets are also endowed with the ability to prevent anoikis (detachment-induced apoptosis) in different human ovarian cancer cell lines by activation of the YAP1 signaling pathway. RhoA controlled YAP-1 gene expression is also responsible for enhanced metastasis [44]. (For an overview of the metastatic cascade see Fig. 1).

### Platelet mediated vascular tumor cell arrest

Beside protection from immune surveillance and anoikis, platelets and platelet adhesion receptors exhibit further beneficial effects for tumor cell adhesion and extravasation to distant organs. Platelet membranes are endowed with a multitude of adhesion molecules like six different integrins (α_IIb_β_III_, α_2_β_1_, α_5_β_1_, α_6_β_1_, α_L_β_2_, α_v_β_3_), GPIb-IX-V, GPVI, CLEC-2, and P-selectin [45]. Most data dealing with the role of adhesion receptors in the metastatic cascade are available for integrin α_IIb_β_III_, GPIb-IX-V, and P-selectin, respectively, their prometastatic activity obviously correlates either with their expression on platelet surfaces [46] or their roles as target molecules in thrombosis and coagulation [45, 47]. The selectin family of adhesion receptors consists of 3 C-type lectins, whereas P-selectin is primarily expressed in platelets as well as endothelial cells [48, 49]. Upon platelet activation, P-selectin from α-granules is translocated to platelet surfaces and mediates platelet binding to leukocytes or endothelial cells, presenting glycan structures like sialylated and fucosylated oligosaccharides for example the tetrasaccharide sialyl Lewis^x^ or its isomer sialyl Lewis^a^. These carbohydrates are terminally clustered on glycoproteins to enhance Ca^2+^-dependent P-selectin binding affinity since P-selectin affinity to isolated sialylated and fucosylated saccharides is quite low [50]. Inhibition of P-selectin blocked accumulation of leukocytes in a growing thrombus as well as fibrin deposition displaying P-selectins’ crucial role in coagulation and leukocyte recruitment [51]. In different studies, a P-selectin blockade or deficiency led to a tremendous reduction in metastatic foci of colon cancer cells in the lungs of mice and a mitigated binding of platelets to tumor cells [52–54]. Binding of LS174T colon adenocarcinoma and THP-1 monocytoid cells to human umbilical vein endothelial cells (HUVECs) under flow was doubled in the presence of platelets, obviously mediated by platelet expressed P-selectin [55].

Furthermore, a differentiation between endothelial and platelet-expressed P-selectin revealed that both, platelet as well as endothelial expressed P-selectin likewise contribute to tumor cell dissemination [56, 57]. Platelets increased number of metastatic nodules in the lungs whereas liver metastasis was surprisingly enhanced in the absence of platelets. These results clearly demonstrate that the mechanism of P-selectin mediated clot formation goes far beyond a simple adherence to the endothelium or getting stuck in small vessels. Becker could show that a ligation of B16F10 melanoma cells to P-selectin induces a p38 MAPK signaling in platelets subsequent to secretion of acid sphingomyelinase, which is pivotal for cancer cell trapping in the lungs [58]. Wang could expose a P-selectin mediated activation of α_IIb_β_III_ through intracellular talin 1 in platelets. The engagement of the P-selectin-α_IIb_β_III_-talin complex led to a deposition of platelets in tumors and finally tumor growth and angiogenesis [59]. P-selectin binding to ligands can also activate pro-survival kinases for instance in neuroblastoma cells that results in enhanced tumor growth in mice [60].

Furthermore, platelets which bind to colorectal cancer cells by means of P-selectin in the presence of polymorphonuclear leukocytes were able to activate human microvascular endothelial cells, which in turn expressed inflammatory proteins, e.g., chemokine CCL5 (Rantes) most abundantly [61]. CCL5 recruited monocytes to the metastatic microenvironment that finally culminated in an augmented number of metastatic foci in the lungs of mice. These data exhibit that platelets and platelet-derived P-selectin seem to be crucially involved in cancer immunity. This is corroborated by findings that P-selectin binding to PSGL-1 on leukocytes induced a recruitment of neutrophils and monocytes to sites of extravasation in small vessels [62]. P-selectin initiates an upregulation of β_2_ integrin affinity in neutrophils. P-selectin-mediated binding of platelets to monocytes induced an adhesive monocyte phenotype with increased clustering and affinity of integrins α_4_β_1_ and α_M_β_2_ to endothelial adhesion molecules and elevated transendothelial migration [63]. Thus, a P-selectin blockade on platelets does obviously not solely comprise a single reduction of tumor cell platelet agglomerate formation; moreover, several protumorigenic and cancer immunological relevant mechanisms can be addressed as well.

Platelet integrins α_IIb_β_III_ and α_2_β_1_ are most relevant for platelet adhesion to proteins of the extracellular matrix and aggregation [45]. GPVI together with integrin α_2_β_1_ mediates firm adhesion of platelets to different collagen types [64]. Notwithstanding, GPVI seems to be mainly responsible for signaling and platelet activation rather than for adhesive roles to collagen, which are subsequently mediated by α_2_β_1_ [65, 66]. The contribution of platelet-derived α_2_β_1_ in the hematogenous metastatic cascade is currently vague, whereas the role of tumor cell-expressed α_2_β_1_ was investigated for prostate cancer cells [67, 68]. This is reasonable since the activity and surface antigenicity of α_2_β_1_ from healthy subjects were found to vary interindividually [69].

Integrin α_IIb_β_III_ is the central platelet receptor that crosslinks adjacent platelets into hemostatic aggregates after trauma or injury. Additionally, it is of crucial importance in the pathogenesis of arterial thrombotic disorders. In resting platelets, α_IIb_β_III_ is in an inactive conformation unable to bind to macromolecular ligands like fibronectin, fibrinogen, vWF, thrombospondin, or vitronectin [64, 70–73]. After platelet activation, α_IIb_β_III_ can switch to at least one of two different active ligand binding states, both differ in their affinity for fibrinogen [70]. In several different studies, the contribution of α_IIb_β_III_ to tumor cell platelet interaction and aggregation was demonstrated [74–76]. Melanoma cell induced platelet aggregation was prone to mAbs directed against α_IIb_β_III_ [77]. A α_IIb_β_III_-induced platelet activation could also be unveiled for glioblastoma and neuroepithelioma cells in vitro [75]. Using platelets with different disorders like Bernard-Soulier syndrome or Glanzmann’s thrombasthenia, a α_IIb_β_III_-dependent platelet aggregation by human osteosarcoma cells could be revealed in which α_IIb_β_III_ initiated a first aggregation which was sustained in a second phase by GPIb-IX-V [76]. Applying a small-molecule α_IIb_β_III_ antagonist administered either orally or intravenously before injection of Lewis lung carcinoma cells, a pronounced reduction of metastatic nodules could be observed in the lungs of mice. These data clearly confirmed the prometastatic role of α_IIb_β_III_ in vivo [78]. Later on, an integrin α_IIb_β_III_-mediated release of proangiogenic factors (vascular endothelial growth factor (VEGF) or basic fibroblast growth factor (bFGF)) was demonstrated due to melanoma or breast cancer cell interaction [79, 80]. These results provided first evidence that α_IIb_β_III_ possesses additional roles in the metastatic spread beyond adhesion and bridging functions. Lonsdorf and colleagues recently revealed a direct binding between platelet α_IIb_β_III_ and melanoma cell-expressed α_ѵ_β_III_ without assistance of fibrinogen or other molecules. Juxtacrine signaling between α_IIb_β_III_ and α_ѵ_β_III_-enhanced lung tumor burden of mice injected with B16-luc melanoma cells [81]. Hence, integrin α_IIb_β_III_ is for several reasons an attractive target in hematogenous cancer cell dissemination. First, different drugs, which efficiently inhibit integrin α_IIb_β_III_, have been approved for reduction or prevention of thrombotic cardiovascular events (e.g., tirofiban, eptifibatide and abciximab). Second, integrin α_IIb_β_III_ is capable of mediating bidirectional signaling. Binding of integrin α_IIb_β_III_ to tumor cells can finally culminate in platelet activation; vice versa, platelet activation by, e.g., ADP, TXA2, or thrombin, can transfer α_IIb_β_III_ to an active binding state. These inside-out signaling confers the ability to bind several ligands for example melanoma cell expressed α_ѵ_β_III_ which can induce protumorigenic and proangiogenic signals [82–84].

Recently, Mangin et al. elucidated an interaction between platelet integrin α_6_β_1_ and tumor cell-derived ADAM9 [85]. ADAM9 is a member of the disintegrin and metalloproteinase (ADAM) family of proteins and consists of a prodomain, a metalloproteinase domain, a disintegrin-like domain, and a cytoplasmic tail and is crucially involved in several cellular processes like proteolytic shedding of membrane-associated proteins [85, 86]. Binding between α_6_β_1_ and ADAM9 initiated platelet activation, P-selectin exposure to the platelet membrane, and finally elevated mammary carcinoma cell transmigration. These data clearly identify integrin α_6_β_1_ as a new potential target on platelets involved in cancer metastasis since several different tumor entities express ADAM9.

GPIb-IX-V is exclusively expressed on platelets and megakaryocytes and belongs to the leucine-rich repeats (LLRs) family of proteins. After vascular injury, GPIb-IX-V mediates the first tethering of platelets to subendothelial vWf even under conditions of high shear rates like in arteriols or small arteries [87]. It consists of four different subunits, GPIbα is disulphide connected to GPIbβ and associated with GPIX and weakly linked to GPV [87]. GPIb-IX-V is the second most abundant receptor on platelet membranes with approximately 25,000 copies of GPIb-IX complexes and 12,000 molecules of GPV per platelet [88, 89]. Next to vWf, thrombin [90], P-selectin [91], leukocyte integrin α_L_β_2_ [92], high molecular weight kininogen, and clotting factors XI and XII have been identified as GPIb-IX-V ligands [93–95].

Upon vWf binding to GPIb-IX-V, activation of phospholipase C, phospholipase A2, and phosphatidylinositide 3-kinases (PI3K) among other kinases is initiated either in cooperation with or independent from Fc receptor FcγRIIA or FcRγ chain [96]. Subsequently, activated protein kinases or second messengers participate in downstream activation of integrin α_IIb_β_III_. In first in vitro experiments, a preincubation of platelets with antibodies to GPIb revealed a mitigated aggregation due to glioblastoma, neuroepithelioma, or breast cancer cell addition [74, 75, 97]. Jain et al. exhibited a prometastatic role for GPIb-IX-V in vivo applying B16F10.1 murine melanoma cells either to wild type C57BL/6J control mice, mice with GPIb-IX-V deficiency mimicking the Bernard-Soulier syndrome, mice with a few residues of GPIb fused to IL-4 receptor (with mild macrothrombocytopenia), or mice lacking six terminal residues of the GPIbα subunit [98]. A 15-fold reduction in number of metastatic foci could be detected in GPIb-IX-V-deficient mice and mice with fused IL-4 receptor to GPIb residues. In contrast, mice with the six residue truncation of GPIbα revealed a moderate but not significant reduction in number of lung metastases compared to wild type mice. These results clearly demonstrate a contribution of GPIb-IX-V to melanoma lung metastasis whereby the GPIb-IX-V mediated signaling seems to be dispensable [98]. Later on, Erpenbeck and colleagues blocked GPIbα with antibody Fab fragments 2 h prior B16F10 cell injection, simultaneously or 2 h after tumor cell inoculation and surprisingly observed a significant increase in number of metastatic nodules in the lungs compared to mice treated with control mabs either simultaneously or previously to tumor cell application [99]. In P-selectin knockout mice, GPIbα had no elevating effect on metastasis. Thus, the role of GPIb-IX-V in the early phase of melanoma metastasis is ambiguous and warrants further investigations maybe in GPIbα knockout mice with spontaneous tumor development.

### Recruitment of neutrophils/monocytes and tumor cell extravasation

Several clinical and experimental investigations have exhibited and elucidated the role of myeloid cells in tumor onset, progression, angiogenesis, and finally local immune suppression [100, 101]. Obviously, also in the early phase of hematogenous metastatic dissemination, the presence and recruitment of myeloid cells seems to initiate and predetermine the metastatic niche formation [102–104].

Agglomerates of platelets and MC38 colon cancer cells have the ability to accumulate CD11b^+^ MMP9^+^ Ly6G^+^ granulocytes via secretion of chemokines CXCL5 and CXCL7 in their vicinity [105]. Both CXCL5 and CXCL7 are released due to platelet activation and attract granulocytes by activation of the granulocyte receptor CXCR2. CXCL5 and CXCL7 release is either initiated by direct contact between platelets and tumor cells or by activation of the coagulation cascade [106]. The prometastatic interplay between platelets, tumor and myeloid cells is corroborated by earlier publications [107]. The presence of leukocytes in tumor cell platelet microthrombi apparently facilitates tumor cell extravasation, supports tumor growth and proliferation, and maybe also participates in local T cell mediated immune suppression [108–110]. It is supposed that platelets have characteristics of immune cells since they share a lot of features with leukocytes and are also involved in a lot of inflammatory diseases like atherosclerosis [111]. Since their role and mechanisms of leukocyte recruitment in inflammatory processes are quite well investigated, platelets’ contribution to metastasis by recruitment of granulocytes to tumor cells and modulation of local immune surveillance as aforementioned is barely explored and will likely offer novel therapeutic options.

### Contribution of platelets to tumor growth and angiogenesis

Upon platelet mediated arrest of the tumor cell embolus at the vascular wall, platelet dense granules secrete ATP, which in turn binds to and activates endothelial P_2_Y_2_ receptors. Subsequently, the endothelial barrier is opened and tumor cell transmigration and extravasation is facilitated to form metastatic foci [112]. Next to ATP, the platelet secretome consists of a plethora of more than 300 bioactive molecules which is sequestered into the local microenvironment after activation [113]. Some of these molecules are endowed with the capability to boost tumor cell proliferation and tumor growth. In an orthotopic pancreatic tumor mouse model, daily administration of P_2_Y_12_ inhibitor (Clopidogrel) decreased tumor growth rate and number of metastatic foci significantly [114]. Blockade of integrin α_IIb_β_III_ resulted in reduced migration, invasion and proliferation of endothelial cells, and also the mean tumor volume of subcutaneous injected melanoma cells into nude mice and rats was profoundly mitigated [115].

Ovarian cancer cells injected into the peritoneal cavity of female nude mice exhibited increased tumor weight due to platelet transfusion and reduced cleaved caspase-3 as marker for apoptosis. In turn, platelet transfusion to tumor bearing mice could rescue the anti-tumor effect of docetaxel administration [116]. In vitro, coculturing of different ovarian cancer cells with platelets, either direct or indirect through a membrane, decreased apoptosis after docetaxel addition [116]. Hence, platelets secreted biologically active molecules exert proliferative and anti-apoptotic signals in various tumor models.

The role of platelets in angiogenesis has long been recognized in several in vitro and in vivo studies applying diverse angiogenic assays [117]. Several molecules, stored in platelets’ α-granules exhibit proangiogenic properties like VEGF [118], platelet-derived growth factor (PDGF) [119], bFGF [120], or epidermal growth factor (EGF) [121] but also molecules exhibiting antiangiogenic effects, e.g., endostatin [122], angiostatin [123], PF4 [124], or thrombospondin [125] among others are located in platelets. Serum VEGF levels of patients suffering from diverse cancers are often elevated which correlates with advanced disease and poor prognosis [126, 127]. Increased VEGF concentrations could potentially contribute to tumor vessel formation, additionally endothelial cells in tumor tissues have the ability to capture platelets by augmented TF expression. Thus, TF finally generates thrombin and activates platelets via PAR-1, which culminates in local platelet settling and growth factor release [128]. Furthermore, Janowska-Wieczorek described PMP which stimulated lung cancer cell to express increased levels of VEGF, hepatocyte growth factor, and IL-8 mRNA and thus may contribute to neovessel formation [129]. Brill et al. could reveal the relevance of PMP for angiogenesis in vivo by transplantation of PMP containing agarose beads subcutaneously into mice [130]. In addition to soluble factor or PMP secretion, upon activation, platelets express CD154, also known as CD40 ligand, which is also capable to expedite angiogenesis [131]. CD40-deficient mice exhibit an impaired tumor growth and an atrophied tumor vasculature in the mammary glands. Similar results were obtained by application of Clopidogrel to mice, which provided evidence, that platelet expressed CD40L and subsequently a direct interaction between platelets and tumor tissue account for neovascularization [132]. As aforementioned, platelets contain pro- and antiangiogenic factors, which are stored in different α-granules and which are released by different stimuli. In the context of tumor cell induced platelet activation and formation of a permissive metastatic microenvironment, the role of different activation pathways are incompletely elucidated. Stimulation of PAR-1, GPVI, or P_2_Y_12_ induced a VEGF release whereas PAR-4 activation or TXA2 instigated an increased endostatin release from α-granules [117, 133–135]. Jonnalagadda and colleagues conducted a more systemic approach and quantified 28 α-granules proteins upon platelet activation with different stimuli and detected a slower release rate and fewer proteins for PAR-4 activation [136]. Preincubation of platelets with heparin or the selective estrogen receptor modulator tamoxifen shifted the release characteristics from a pro- to an antiangiogenic phenotype with attenuated VEGF release subsequent to MCF7 breast cancer cell interaction [137, 138]. These findings are in line with recent data from Peters who distinguished different platelet granule subpopulations by VAMP (vesicle-associated membrane protein) isoform expression [139].

Due to the fact that platelets contain different MicroRNAs, which are release in PMP upon platelet activation, it is tempting to speculate that MicroRNAs contribute to neovascularization in growing tumors as well. PMP from patients with myocardial infarction for instance mediated the transfer of MicroRNA to endothelial cells [140]. Thus, PMP-derived MicroRNAs are capable to regulate gene expression in endothelial cells and could potentially impact neovascularization.

Angiogenesis is not limited to local expansion of vessels under the impact of growth factors. Additionally, the recruitment of bone marrow-derived progenitor cells sustains the formation of neovessels. Feng and colleagues could illustrate that platelet VAMP-8 containing α-granules mobilize and recruit bone marrow-derived cells to sites of hypoxic stress and tumor tissue. Finally, bone marrow-derived cells contribute to formation of new vessels in the tumor tissue [141].

Platelets facilitate angiogenesis in many different ways but the precise mechanisms of platelet granule secretion of different proangiogenic and antiangiogenic factors are only partially elucidated. A deeper insight in platelet signaling pathways, involved receptors, and granule organization will offer new therapeutic opportunities for manipulation in platelet mediated support of angiogenesis.

## Involvement of platelet ITAM and hemi ITAM receptors in cancer metastasis

### CLEC-2 and the interaction with podoplanin

CLEC-2 was initially identified as transcript in monocytes, dendritic cells, NK, and endothelial cells [142, 143]. Later on, CLEC-2 expression was perceived on platelets as the ligand for the potent platelet activating snake venom protein rhodocytin [144]. CLEC-2 is a 32 kDa type II transmembrane glycoprotein with an extracellular C-type lectin-like domain. Intracellularly, CLEC-2 contains a hemi ITAM (hemITAM) YxxL motif in its cytoplasmatic tail, which is similar to ITAM motifs comprising a YxxLx(6–12)YxxL sequence [145, 146]. Upon CLEC-2 ligand binding or antibody-mediated dimerisation, Src phosphorylates hemITAM and SH2 domain-containing tyrosine kinase Syk which in turn conducts additional hemITAM motif phosphorylation and autophosphorylation [147–149].

**Fig. 2:**
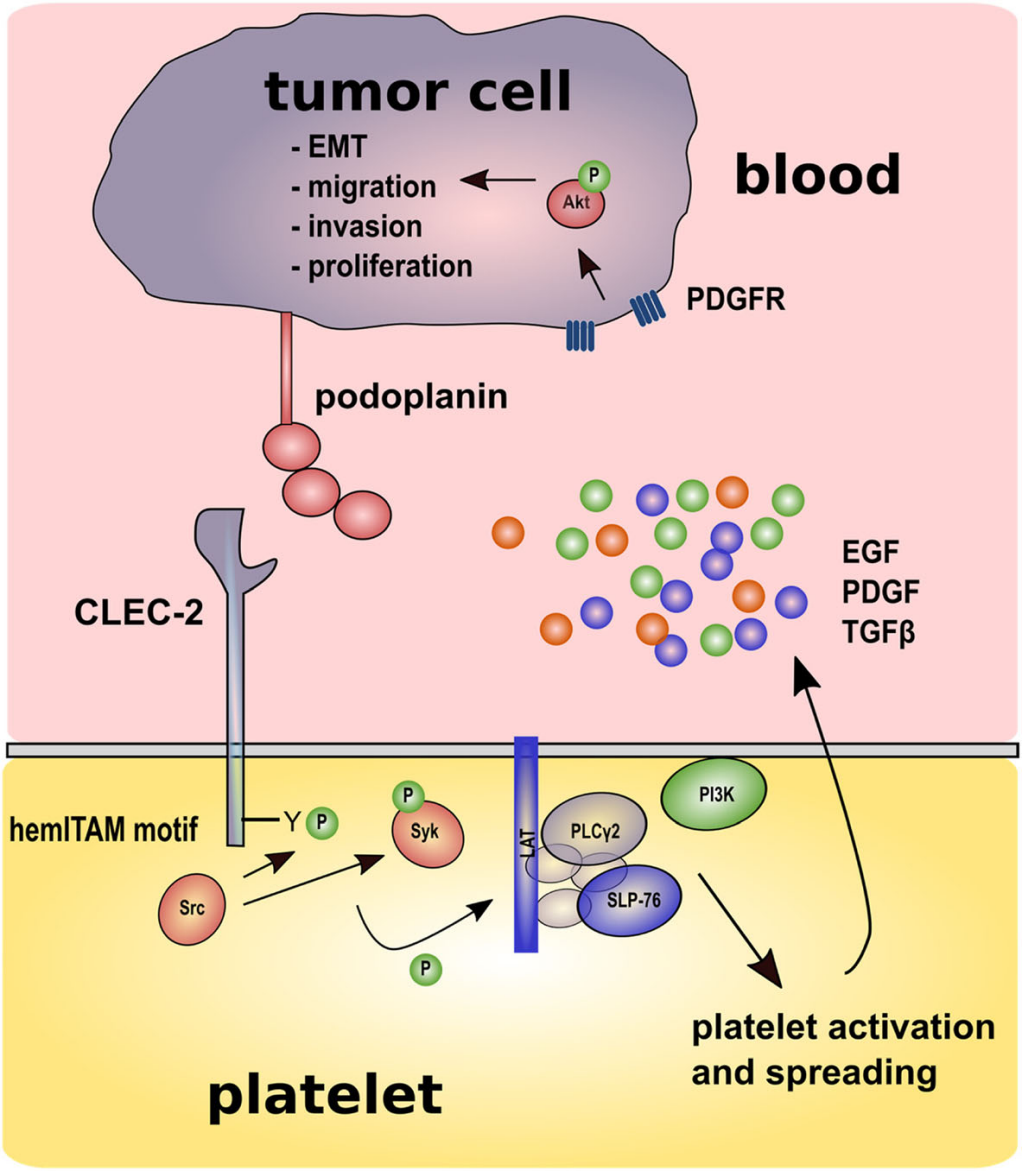
Contribution of CLEC-2 to platelet tumor cell cross-talk. Tumor cells expressing podoplanin interact with CLEC-2 localized on platelets. Upon podoplanin ligation, hemITAM motif and Syk are phosphorylated by Src. Activated Syk in turn phosphorylates hemITAM, Src and a signaling complex consisting of LAT, SLP-76 and PLCγ2. Finally, PI3K activation leads to platelet activation and secretion of growth factors, e.g., EGF, PDGF, and TGF-β. PDGF induces Akt phosphorylation ensued by osteosarcoma cell proliferation. TGF-β conducts a shift from an epithelial to a mesenchymal phenotype accompanied by increased tumor invasiveness and proliferation

Signaling downstream from Syk is nearly identical to that of ITAM motif containing receptors. Syk phosphorylates and activates linker for activation of T cells (LAT) and SH2 domain-containing leukocyte-specific protein of 76 kDa (SLP76) to form a signaling complex culminating in activation of PI3K and phospholipase Cγ2 and finally platelet activation and spreading (Fig. 2). Watanabe and colleagues identified a membrane protein in highly metastatic NL-17 mouse colon carcinoma cells, which induced a strong platelet aggregation in the absence of plasma components. Antibody-mediated inhibition of this proaggregatory protein on NF-17 cells reduced pulmonary metastasis in vivo [150, 151]. This protein, designated as Aggrus, was later assigned to be identical with the lymphatic endothelial marker podoplanin [152, 153]. In 2007, podoplanin was identified as CLEC-2 ligand responsible for pronounced platelet aggregation and a podoplanin recognition domain in CLEC-2 was elucidated [154–156]. Podoplanin contains three platelet aggregation-stimulating domains in the extracellular section, O-glycosylated on threonine residues, which is crucially for the aggregating function [157]. In healthy human tissues, podoplanin is expressed in lymphatic endothelial cells, podocytes, osteocytes, keratinocytes, and myofibroblasts among many other cell entities [158]. Mice deficient in podoplanin reveal defects in lymphatic vessel formation and die after birth due to respiratory failure [159].

Also in different types of cancers, for instance in squamous cell carcinoma, lung and skin cancer, mesotheliomas, and cancer-associated fibroblasts (reviewed in [160, 161]), an upregulated expression of podoplanin was detected. Podoplanin induced an epithelial to mesenchymal transition (EMT) in MDCK cells, increased cell migration, and was associated with tumor invasion [162]. Meanwhile, the tumor cell-induced platelet aggregating effect of the podoplanin CLEC-2 interplay was revealed in several animal models and different antibodies targeting different epitopes were generated [163, 164]. The number of metastatic nodules in the lungs of popoplanin transfected CHO or HT1080 fibrosarcoma cells was reduced by antibody-mediated podoplanin blockade [164]. Kunita created CHO cells expressing podoplanin and could detect an arrest in the lung microvasculature 30 min after injection in mice. Additionally, number of metastatic nodules was increased by mice injected with podoplanin-expressing cells compared to wild type cells. The size of the primary tumor or metastases was not affected by popoplanin presence. Applying podoplanin point mutants, unable to bind CLEC-2, reduced platelet aggregation and finally metastasis formation [165]. Takagi et al., in turn, could also detect an effect of anti-podoplanin antibodies on primary tumor growth of lung squamous cell carcinoma PC-10 cells accompanied by strong reduction of metastatic foci in the lungs [166]. For osteosarcoma cells, a podoplanin-mediated platelet activation led to a PDGF receptor (PDGFR) signaling ensued by Akt phosphorylation which fostered osteosarcoma cell proliferation [167]. Recently, Takemoto revealed podoplanin-induced platelet secretion by bladder squamous cell carcinoma cells. Aggregated platelets released TGF-β, which in turn induced an EMT in carcinoma cells and enhanced invasiveness and metastatic spread. A TGF-β blockade attenuated tumor extravasation and pulmonary metastasis [168]. These data are in accordance with Labelle and colleagues who indicated a mesenchymal-like phenotype for breast cancer cells previously treated with platelets [169]. Nonetheless, the platelet-induced effects on invasion and metastasis require TGF-β and an additional direct juxtacrine interaction between platelets and breast cancer cells. Both signals obviously synergize to confer a mesenchymal and stem cell-like phenotype to breast cancer cells [169]. Studies, dealing with a targeted inhibition of CLEC-2 to hamper podoplanin-mediated platelet activation in hematogenous dissemination are hardly available. Weng et al. recently reported on a small-molecule inhibitor of CLEC-2, which inhibited podoplanin-mediated platelet activation and increased survival time of mice in combination with cisplatin treatment [170]. Bleeding risk of mice, in turn, was not affected by application of this novel CLEC-2 inhibitor. These results were corroborated by Shirai and colleagues by depleting CLEC-2 with antibodies from platelet membranes [171]. Mice injected with B16F10 melanoma cells and deficient in CLEC-2 revealed a significantly prolonged overall survival which may be due to reduced thrombus formation in the lungs. Hence, CLEC-2 is an attractive target to circumvent CLEC-2 podoplanin-mediated platelet activation and aforementioned protumorigenic effects. Nonetheless, other studies report of an antibody-mediated ligation of CLEC-2, which was found to induce CLEC-2 immunodepletion accompanied by a severe transient thrombocytopenia [172]. Recently, Lorenz et al. revealed a CLEC-2 internalization from platelet membranes by application of anti-CLEC-2 antibody. This process depends on Src activity, which is downstream associated with hemITAM phosphorylation, but is surprisingly independent from Syk activation. A blockade of Src activity prevented CLEC-2 internalization. In wild type mice, platelet activation was observed followed by platelet clearance from the circulation. In contrast, utilizing Syk-deficient mice, an antibody-mediated CLEC-2 internalization could be detected whereas a platelet activation, CLEC-2 clearance, and thrombocytopenia were finally prevented [173, 174]. These results offer the intriguing opportunity for a combination therapy of CLEC-2 antibody internalization and Syk inhibitor mediated perpetuation of CLEC-2 denuded platelets in the circulation.

Thus, the CLEC-2 podoplanin axis is an interesting target in course of hematogenous metastasis especially for those patients with podoplanin-positive tumors. However, more studies and data are eligible to elucidate the physiological implications of a long-term podoplanin or CLEC-2 inhibition, respectively.

### Contribution of GPVI and FcγRIIa to platelet activation, EMT, and metastasis

**Fig. 3:**
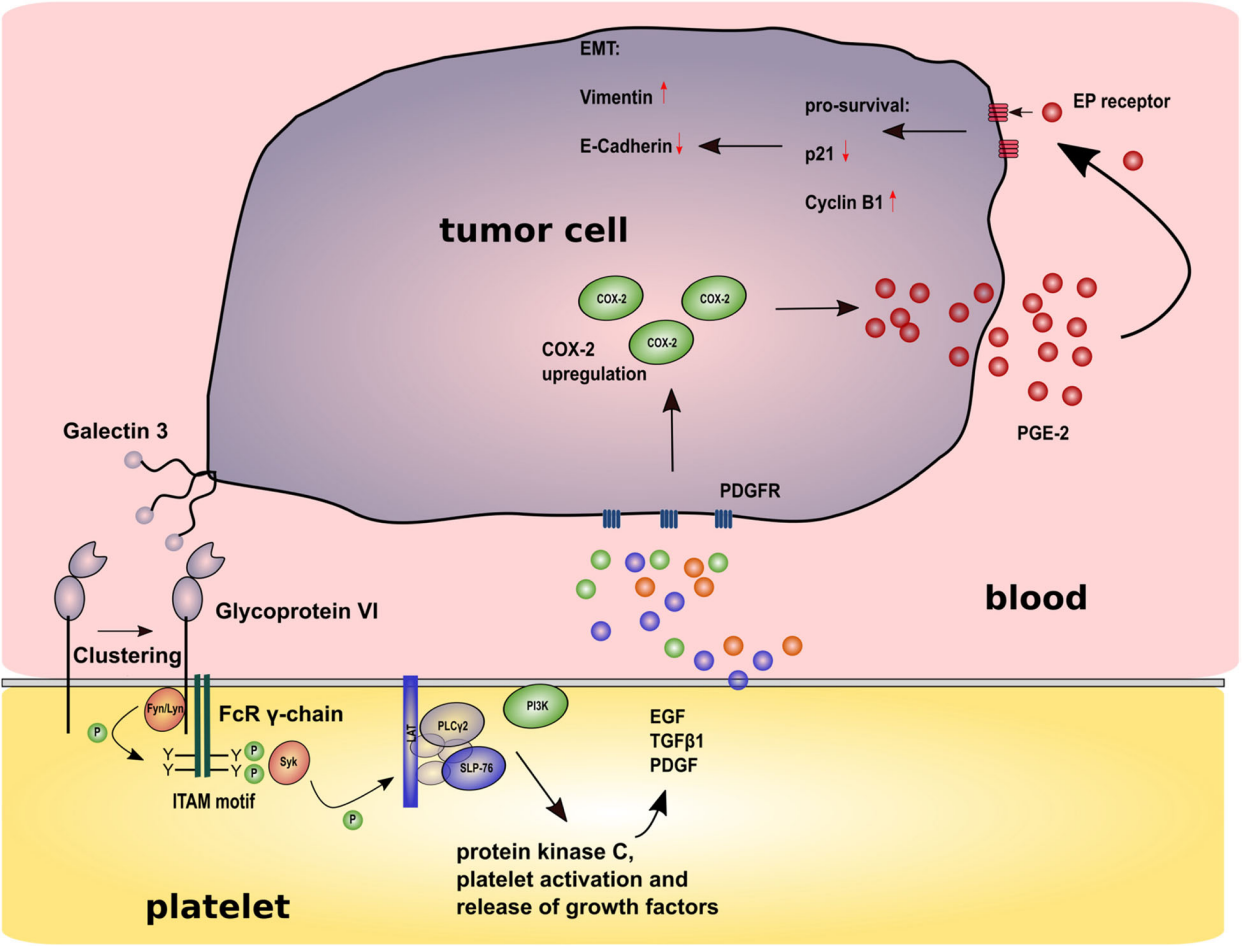
Impact of the interaction between GPVI and galectin 3 on cancer metastasis. Galectin 3 on colon cancer cells binds to GPVI on platelets which induces GPVI clustering and ITAM phosphorylation by Fyn and Lyn kinases. Syk and finally platelet activation induces discharge of growth factors like PDGF, EGF, and TGF-β1 among others. PDGF activates PDGFR inducing an upregulation and stabilization of COX-2 and release of PGE_2_ from tumor cells. PGE_2_ in turn binds to tumor cell EP receptors, which leads to p21^waf^ downregulation and an upregulation of cyclin B1. Additionally, a deregulation of EMT-related marker proteins is detectable with Vimentin up- and E-Cadherin downregulation

GPVI is exclusively expressed on platelets and megakaryocytes and was initially identified in a patient with platelets deficient in collagen induced aggregation and adhesion [175, 176]. GPVI is a transmembrane protein with an extracellular domain consisting of two immunoglobulin C2-like domains, orientated at 90°, a stalk, a transmembrane domain, and a cytoplasmatic tail [177]. In areas of vascular injury and subendothelial matrix exposure, GPVI in conjunction with integrin α_2_β_1_, is the key receptor for collagen type I and responsible for stable platelet adhesion after a transient interaction of GPIb-IX-V with vWf at high shear rates [65, 66, 177]. A density of approximately 4000 copies of GPVI per platelet was reported, partially organized in a mono- or dimeric form in tetraspanin microdomains regulating GPVI lateral diffusion [178–180]. Upon binding of GPVI dimers to collagen, a clustering of GPVI was described accompanied by increased avidity towards collagen and enhanced proximity of GPVI associated signaling molecules [181, 182]. GPVI cytoplasmatic tail is associated through a salt bridge with the Fc receptor γ chain (FcRγ) and upon ligand mediated GPVI crosslinking and clustering, ITAM motif in FcRγ chains are unmasked and phosphorylated by Src kinases Lyn and Fyn (Fig. 3). Phosphorylated ITAM in turn recruits and activates Syk, which assembles downstream a signaling complex composed of LAT and SLP76. This complex finally leads to platelet activation and spreading [183, 184].

The role of this ITAM containing receptor in the interaction of platelets with tumor cells is hardly investigated and just a few studies are currently available shedding light on this issue. Jain and colleagues generated C57BL/6J mice deficient in GPVI with prolonged bleeding time and reduced thrombus formation [185, 186]. Induction of primary tumors in the dorsal flank of these mice revealed no differences between wild type and GPVI-deficient mice in tumor size and number of tumor microvessels. In experimental metastasis approaches, in contrast, number of metastatic foci in the lungs was significantly reduced for B16F10 melanoma as well as for D121 mouse Lewis lung carcinoma cells, respectively [187]. These in vivo experiments figured out the contribution of GPVI to the metastatic spread of different tumor cell lines whereas the exact mechanism by which GPVI supports metastasis remained ambiguous. Later on, Dovizio explored the mode of action by which platelet GPVI fosters protumorigenic properties of human colon cancer in an in vitro approach. They could unravel an interaction between GPVI on platelets and colon cancer cell expressed galectin 3, which induced release of different growth factors from platelet granules like EGF, TGF-β1, and most abundant PDGF (Fig. 3) [188]. Binding between GPVI and galectin 3 together with PDGF initiated a cyclooxygenase-2 (COX-2) upregulation and stabilization in colon cancer cells and finally a release of increased PGE_2_ levels from tumor cells with a maximum after 20 h of platelet tumor cell interaction. Blocking galectin 3 reduced COX-2 induction in colon cancer cells significantly. Discharge of PGE_2_, in turn, triggered via EP receptors in an autocrine fashion a downregulation of apoptosis and proliferation associated protein p21^waf^ and an upregulation of cyclin B1. Finally, epithelial and mesenchymal marker proteins in colon cancer cells were quantified and an induction of ZEB1, Twist1, and vimentin was detectable, whereas E-cadherin was downregulated. This effect was sensitive to a selective COX-2 inhibitor treatment, illustrating that GPVI induced COX-2 expression is ultimately responsible for colon cancer cell transition from an epithelial to a mesenchymal phenotype adopting cancer stem-like properties [189–191]. Thus, next to collagen [176, 177, 192], fibronectin [193], fibrin [194], and laminin [195], galectin 3 seems to be an additional ligand for GPVI relevant for platelet activation and vice versa for an increase in tumor cell malignancy. COX-2 expression in cancer cells seems to be a general driver of cancer progression and especially lymphangiogenesis. In one study, a celecoxib-mediated inhibition of COX-2 reduced podoplanin expression in breast cancer tumors [196]. Kubo and colleagues detected a COX-2 and EP3 receptor-dependent lymphangiogenesis with podoplanin as marker for lymphatic vessels in model of Lewis Lung cells [197]. This is in line with Hosono et al. who detected COX-2 dependent lymphangiogenesis in a Matrigel assay with fibroblast growth factor-2. Podoplanin was utilized as marker for lymphatic endothelial cells [198]. Shen et al. could detect a galectin 3 downregulation in COX-2 overexpressing mice which were finally refractory for tumor-promoting agents [199]. Thus, COX-2, podoplanin and galectin 3 are obviously intertwined in their support to tumor growth in different fashions and highlight COX-2 as valuable target in lymphangiogenesis.

Besides GPVI, human platelets express the ITAM receptor FcγRIIa, a low-affinity receptor for immune complexes [200]. Mouse platelets on the contrary do not express FcγRIIa [201–203].

**Fig. 4:**
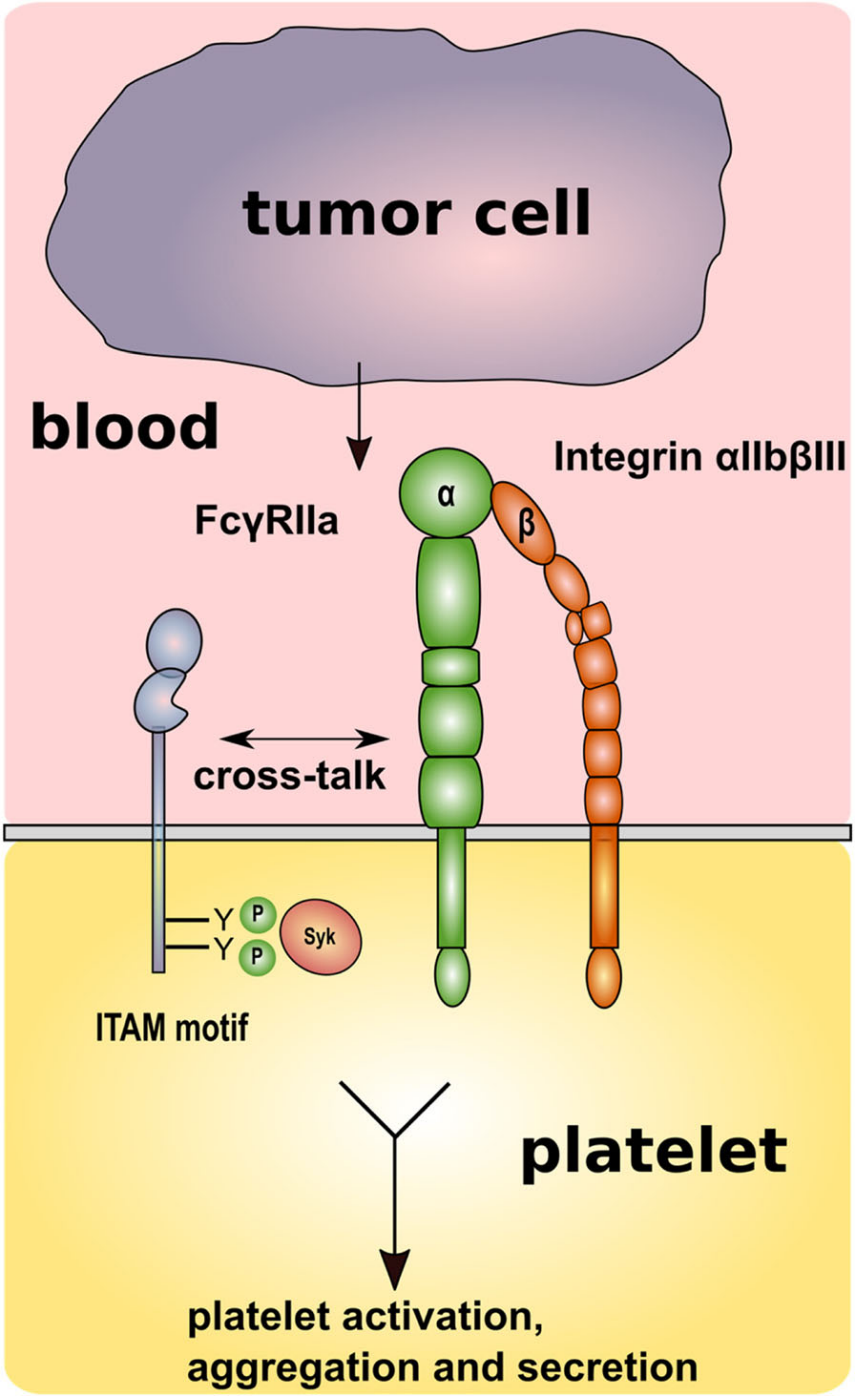
Role of the FcγRIIa α_IIb_β_III_ interaction on tumor cell mediated platelet activation. Prostate cancer cells induce platelet activation exploiting a FcγRIIa and Syk dependent mechanism. Obviously, platelet activation is expedited by a cross-talk between FcγRIIa and integrin α_IIb_β_III_ culminating in platelet aggregation and ADP secretion

The role of FcγRIIa is well investigated in heparin-induced thrombocytopenia as well as platelet activation by IgG-opsonized bacteria. Furthermore, an IgG-independent FcγRIIa-mediated cooperation between GPVI, GPIb-IX-V, and α_IIb_β_III_ for platelet activation and spreading has been suggested. Studies dealing with the participation of FcγRIIa in tumor metastasis are barely available [200]. Mitrugno et al. could recently deduce that FcγRIIa dependent signaling is responsible for PC3M prostate cancer cell mediated platelet ADP secretion ensued by platelet aggregation (Fig. 4) [204]. Inhibition of FcγRIIa downstream signaling molecules like tyrosine kinase Syk, phospholipase Cγ2, or PKC resulted in alleviated platelet secretion and aggregation due to prostate cancer cell contact. The authors propose a collaborative FcγRIIa integrin α_IIb_β_III_ cross-talk as mechanism for FcγRIIa activation and finally ADP secretion, since experiments were conducted in vitro with washed prostate cancer cells and washed platelets, where no exogenous antibodies are present for a potential activation of FcγRIIa. On the contrary, under in vivo conditions, a direct activation of platelet FcγRIIa via Fc portions of anti-tumor antibodies is conceivable [205]. In further studies, it is mandatory to include several tumor cells from different entities to elucidate their mechanisms of platelet secretion and aggregation.

## Conclusion

Platelets contribute to cancer progression, metastasis and angiogenesis in multiple different ways.

The role of ITAM or hemITAM comprising receptors in platelet-assisted metastasis became more evident since the discovery of podoplanin as CLEC-2 ligand and Galectin-3 as newly identified ligand for GPVI, respectively. Hence, the understanding of these receptors in metastasis will accelerate in the future and maybe reveal as valuable new therapeutic targets for interference in hematogenous metastasis as well as in prevention of thrombosis.
